# Common factors and nutrients affecting intestinal villus height -A review

**DOI:** 10.5713/ab.25.0002

**Published:** 2025-05-12

**Authors:** Jialu Wang, Yuying Wu, Tiantian Zhou, Yingying Feng, Liu-an Li

**Affiliations:** 1 Tianjin Key Laboratory of Agricultural Animal Breeding and Healthy Husbandry, College of Animal Science and Veterinary Medicine, Tianjin Agricultural University, Tianjin, China; 2 Tianjin Institute of Industrial Biotechnology, Chinese Academy of Sciences, Tianjin, China; 3 College of Animal Science and Technology, Gansu Agricultural University, Lanzhou, China; 4 State Key Laboratory of Quality Research in Chinese Medicine, Institute of Chinese Medical Sciences, University of Macau, Macao SAR, China

**Keywords:** Intestinal Health, Intestinal Morphology, Small Intestinal Villus, Villus Height

## Abstract

The villus of the small intestine plays an essential role in the digestion and absorption of nutrients. They mix chyme with digestive secretions and absorb nutrients by assisting in food agitation and adherence in the intestinal lumen. The height of villi is a critical indicator of the effective absorptive area of the small intestine, which will be greatly reduced if the villi are shortened. Many factors influence the height of intestinal villi, including age, diet, disease, and environmental conditions. This review summarizes the common factors affecting intestinal villus height to provide theoretical guidelines for enhancing intestinal health.

## INTRODUCTION

The small intestine is an essential organ for food digestion and absorption, performing a vital metabolic role in the body [1]. It is characterized by its great length, large area, numerous villus, and multiple types of digestive secretions, which create optimal conditions for absorption, motility and secretion [2]. The small intestine is mainly composed of the lamina propria, intestinal glands, submucosa, internal circular muscles, external longitudinal muscles, and the most specialized structure: the intestinal villus [3,4]. Located on the circular folds of the small intestine, villus appears as a hair-like protrusion into the lumen [5]. Notably, the villus and microvillus increase the absorptive area of the small intestine by about 600-fold, reaching 200 to 250 m^2^, which allows them to help stir food in the intestinal lumen for adequate digestion and absorption of nutrients [6]. Furthermore, the small intestinal villus are enriched with capillaries, lymphatic capillaries, smooth muscles and a nerve fiber network [7]. The growth of villus requires complex and tight epithelial-mesenchymal interactions, which are regulated by signaling molecules such as hedgehog, bone morphogenetic protein, physical forces and epithelial deformation [8].

Intestinal villus height is a key indicator of the effective absorption area of the small intestine. Generally, a larger absorption area provides a greater capacity to take up nutrients [9]. Therefore, the height of intestinal villus directly affects the absorption of nutrients in the small intestine.

## EFFECT OF DIET ON INTESTINAL VILLUS

There is substantial evidence that nutrient intake leads to changes in intestinal morphology and remodeling of the intestinal tract. This process involves changes in the length of the intestine, as well as the growth of small intestinal villus and microvillus, which alters energy absorption to maintain energy homeostasis [10].

### Nutrient intake

Nutritional deficiencies lead to gastrointestinal disturbances such as loss of appetite, nausea, vomiting, bloating and diarrhea. Relatedly, malnutritional enteropathy is associated with intestinal barrier weakness and immune imbalance [11].

Force-feeding is a useful treatment for animals that are incapable of eating by themselves and are undernourished, which is one of the factors affecting the height of the intestinal villus. A comparison of the effects of force-feeding on the intestinal physiological functions of Gang and Tianfu geese revealed that force-feeding reduced the thickness of the intestinal barrier, increased the height of intestinal villus, and decreased the depth of the small intestinal crypts in Tianfu geese.

On the other hand, restricted feeding or starvation causes changes in villus height and crypt depth, which affects nutrient absorption from the gastrointestinal tract. In the study of Santos et al., the average height of villus on ileal epithelium was shorter in lambs subjected to restricted feeding than in free-feeding lambs [12]. Similarly, early protein restriction in piglets reduced villus height in the jejunum, whereas switching to normal protein reinstated villus height, suggesting intestinal morphology could be improved during the re-nutrition stage [13].

In rabbit husbandry, feed restriction is often used to reduce the incidence of post-weaning digestive disorders such as rabbit enteropathy syndrome and digestive diseases. It was observed that the rabbits in the 65 g/day restriction group had a higher small intestinal villus height than those in the ad libitum and 50 g/day restriction groups, indicating the significant effect of feed restriction on villus height [14]. Another piglet study showed that maternal nutritional deficiencies resulted in shorter jejunal and ileal villus heights in neonatal piglets, so force-feeding or restriction of feeding is assessed on a production-specific basis to ensure that neonatal piglets receive adequate nutrition [15].

### Nutrient content

Vitamin A denotes a diverse group of micronutrients that have beneficial effects on overall health. These compounds regulate biological functions, including vision, growth and regulation of the intestinal barrier [16]. Lack of vitamin A in the diet will cause alterations of commensal bacteria within a few weeks, further compromising the intestinal barrier by modifying mucin dynamics and expression of defense molecules such as MUC2 and defensin 6 [17]. Vitamin A deficiency is also associated with reduced small intestinal villus height and decreased disaccharide activity, leading to more severe intestinal damage in enteritis. Xiao et al showed that villus height in the duodenum of control ducks was markedly lower than in the vitamin A-supplementation group, and duodenal development was severely impaired without vitamin A in the diet [18].

Carbohydrates, comprising sugars and starches, represent the predominant source of calories in the diets of humans and animals. These nutrients provide the body with energy and ensure the proper functioning of bodily systems. It is evident from the study of Zhao et al. that the height of intestinal villus in Pikeperch diminishes in proportion to elevated levels of starch (8%, 10%, 12%) [19]. Similarly, in yellow catfish, vitamin D3 (VD3) and a high carbohydrate diet (HCD) interacted with each other to affect the height of intestinal villus (p<0.05). HCD increased the height of intestinal villus compared to the control group. In contrast, VD3 decreased the height of intestinal villus [20].

In daily diet, it is vital to recognize the indispensable role of protein and fat in the growth, reproduction and maintenance of animals. In the event of diets that are deficient in protein and fat, there is a high probability of adverse effects on the animal’s body functions and reproductive performance, and the gastrointestinal tract is no exception to this. In the study conducted by Incharoen et al. on poultry, a decrease in dietary protein from 18.1% to 9.4% was found to result in lower duodenal and ileal villus height (p<0.05) and the duodenal villus area was reduced (p<0.05) in both the low-crude protein or low-crude fat groups fed a low-protein diet [21]. However, following such low protein feeding, supplementation with certain branched chain amino acids has been demonstrated to restore damaged villus. It has been demonstrated that the small intestine, a vital organ in the digestive and absorptive process, is susceptible to impairment in the absence of adequate protein intake. This, in turn, results in its inability to function optimally in the process of nutrient absorption.

The addition of minerals in the proper and appropriate amounts ensures that the organism can consume enough trace elements, which have important roles in the digestive process. In order to evaluate the effect of complex amino acid minerals (ZMCAA) and bis-glycine chelated minerals (ZMCGLY) on the performance of producing hen dietary pairs, it was found that the source of trace minerals had a significant effect on the villus height of the laying hens (p<0.05). Furthermore, hens fed diets containing ZMCGly exhibited longer fluffs compared to hens fed diets containing ZMCAA. The results of this study demonstrate that the provision of trace minerals can not only enhance mineral intake but also facilitate digestive and absorptive processes [22]. Additionally, in a study to investigate the effect of early dextrose (Dex) feeding on subsequent growth performance of broilers, duodenal villus height at 7 days of age was found to be reduced by Dex feeding in terms of duodenal villus height (p<0.01) [23].

Based on the above research, a reasonable diet and supplementation of the right amounts of nutrients can improve the height of small intestinal villus to maintain the healthy state of the intestinal tract.

## EFFECTS OF FUNCTIONAL NATURAL PRODUCTS ON INTESTINAL VILLUS

Nutrients are diverse in terms of both structures and sources, with equally wide-ranging effects on disease prevention and treatment. Natural products also have the positive effects of immunomodulation, regulating the composition of intestinal flora, and improving intestinal mucosal barrier function [24].

### Functional saccharides

A study in mice fed a high-fructose diet (60% fructose for 8 weeks) demonstrated a 25% increase in jejunal villus height (from 320 μm to 400 μm), accompanied by elevated serum triglycerides (from 120 mg/dL to 180 mg/dL). This was attributed to enhanced lipid absorption and upregulated expression of fatty acid transport proteins in villus epithelial cells [25]. Under hypoxic conditions, fructose 1-phosphate accumulation inhibited pyruvate kinase activity, increasing epithelial cell survival and further promoting villus growth [26]. Similar to fructose, inulin, a prebiotic fiber, was shown to increase villus height in rats by 15% (from 400 μm to 460 μm) when supplemented at 5% of the diet [25]. Similarly, raffinose supplementation in broiler chickens increased villus height by 12% (from 450 μm to 504 μm) and improved nutrient absorption [27].

Chitosan oligosaccharides (COS) are the only naturally occurring positively charged oligosaccharides and are considered functional marine prebiotics. Numerous studies have shown that COS could be used as a novel feed additive to regulate intestinal microecology and improve intestinal tissue morphology in pigs, broiler chickens, rats, and fish, as well as to enhance overall immune function. A study showed that COS supplementation increased villus height by 18% (from 500 μm to 590 μm) and reduced crypt depth by 10% (from 120 μm to 108 μm), leading to an improved villus height-to-crypt depth ratio [28]. Another study reported a dose-dependent increase in villus height, with 200 mg/kg of COS increasing villus height by 22% (from 480 μm to 585 μm) in broilers [29].

Muscovy duck reovirus (MDRV) infection can cause severe immunosuppression and intestinal injury with high morbidity and mortality. Studies have shown that pretreatment with astragalus polysaccharide (APS) can effectively protect ducklings from MDRV infection. It was shown that APS greatly increased the height of MDRV-infected small intestinal villus. This suggests that APS pretreatment can improve intestinal morphology, repair the damage of the mucosal immune barrier in MDRV-infected ducklings, and effectively stimulate the mucosal immune function [30]. *Enteromorpha prolifera* polysaccharide-Fe (III) complex increased duodenal and jejunal villus height in pigs by 25% (from 300 μm to 375 μm), indicating its role in iron fortification and intestinal development [31].

Moreover, some natural polysaccharides also have positive effects on the intestinal tract. For example, fucoidan can protect the intestinal barrier and help repair damage. Guo et al fed fucoidan to weaned lambs and found that the villus height had a significant increase in the treated group compared to the controls, with a concomitant improvement of the intestinal barrier and function of the small intestine [32]. Similarly, *atractylodes* polysaccharide increased villus height by 15% (from 400 μm to 460 μm) in broilers [33], while yam polysaccharides [34] and lentinan [35] showed comparable effects in improving piglets’ intestinal morphology.

### Functional flavonoids

Flavonoids, such as those found in Mori folium, significantly increased intestinal villus height in penaeus vannamei shrimp by 18% (from 200 μm to 236 μm) and promoted intestinal flora diversity [36]. *Andrographis paniculata*, containing diterpenoid lactones and flavonoids [37], increased villus height in ducks by 15% (from 450 μm to 517 μm) and improved nutrient absorption [38].

### Functional oils

Some plant extracts, such as essential oils, have positive effects on intestinal function, while also having antimicrobial, anti-inflammatory, and intestinal flora regulating effects [39]. Oregano oil, both natural and synthetic, increased villus height in broilers by 20% (from 500 μm to 600 μm), improving intestinal health [40]. For weaned pigs, Tea tree oil [41] and chili oil [42] also showed similar effects, with villus height increases of 15% (from 480 μm to 552 μm) and 18% (from 470 μm to 554 μm), respectively. Similarly, adding complex plant essential oils to weaned pigs diets has also been confirmed to increase the height of intestinal villus [43].

### Functional polyphenols

In addition to their well-known antioxidant capacity, polyphenolic compounds found in plant extracts have a wide variety of different effects, such as anti-inflammatory, antibacterial, hypoglycemic and hypolipidemic activities, while also aiding digestion and promoting intestinal health. Curcumin supplementation in broiler ducks increased duodenal and ileal villus height by 25% (from 400 μm to 500 μm), promoting mucosal development [44]. Tannins are complex derivatives of ellagic acid with a polyphenolic hydroxyl chemical structure and related properties. It has been reported that low doses of tannins added to feed showed beneficial effects on animals [45]. Chestnut tannins increased villus height in weaned piglets by 20% (from 350 μm to 420 μm) and reduced diarrhea incidence [46].

### Functional alkaloids

Alkaloids are nitrogen-containing secondary metabolites of plants and major active ingredients of herbal medicine. *Gelsemium elegans alkaloids* increased villus height in the duodenum, jejunum, and ileum of weaned piglets by 18% (from 300 μm to 354 μm), enhancing nutrient absorption [47]. Moreover, sanguinarine, which was isolated from *Macleaya cordata*, was also found to improve intestinal morphology. Sanguinarine remarkably increased villus height by 22% (from 320 μm to 390 μm) in piglets [48].

### Saponins

Saponins are glycosides with triterpenoid or steroidal aglycones, which are mainly found in terrestrial higher plants. One of the most famous saponins is ginsenoside, the potent medicinal ingredient in the herbal composition of ginseng. It was shown that dietary ginsenoside Rg1 dramatically increased jejunal villus height in broilers by 20% (from 450 μm to 540 μm) and improved the crypt-villus ratio while at the same time significantly decreasing the jejunal crypt depth of broilers [49]. Saccharicterpenin, a mixture of polysaccharides and saponins, became the first feed additive approved by the Ministry of Agriculture in China following independent research and development [50], increased villus height in juvenile turbot fish by 25% (from 200 μm to 250 μm) [51]. In addition, a study by Huang et al. found that saccharicterpenin had a promoting effect on the growth of intestinal villus in broilers [52].

### Organic acids

Organic acids are widely distributed in the leaves, roots and especially fruits of plants. They have various effects such as stimulating the secretion activity of digestive glands, improving appetite, as well as assisting digestion and absorption. Compound organic acids increased duodenal villus height in 21-day-old broilers by 15% (from 400 μm to 460 μm) [53]. Using a combination of probiotics and organic acids on weaned piglets significantly increased jejunal and ileal villus height in weaned piglets by 18% (from 350 μm to 413 μm), reducing diarrhea rates [54].

Amino acids in dietary proteins may indirectly influence intestinal morphology by modulating the composition of intestinal microbiota. Adding 0.8% arginine improved the height of intestinal villus in the duodenum, jejunum and ileum of piglets, as well increasing the content of small intestinal mucosal proteins. A positive effect of arginine supplementation on intestinal growth and development was demonstrated in the early post-weaning period. Likewise, supplementation of methionine in the post-weaning diet resulted in higher jejunal villus height in piglets [55]. Sulfur-containing amino acids (SAA) in the diet contribute to optimal intestinal health, so their content in the diet has a direct impact on intestinal villus height. In addition, lysine increased intestinal villus height, and the best SAA: lysine ratio based on intestinal villus height was 60%, 63% and 66%, respectively [56].

### Other compounds

In the feeding process of livestock and poultry, adding the appropriate amounts of probiotics, amino acids, and other non-toxic, residual-free additives can improve the height of small intestinal villus, which can promote nutrient metabolism and digestion, further enhancing the feed utilization rate of animals.

Probiotics are another important group of beneficial substances that are closely associated with intestinal health. Awad et al. found that probiotic chicory, which is rich in inulin, statistically significantly increased the villus height in the duodenum of broiler chickens [57]. Probiotics, such as *Bacillus coagulans*, increased intestinal villus height in silver sillago fish by 20% (from 250 μm to 300 μm) [58]. In addition, it was found that supplementing sows with probiotics or their synbiotic mixtures with prebiotics improved the immune response of their offspring by altering the intestinal flora. The height of jejunal and ileal villus was increased in the probiotic and synbiotic groups compared to the control group. This suggests that increasing the maternal beneficial microbiota through dietary supplementation during gestation and lactation is a potential strategy for regulating the intestinal health of offspring piglets [59].

As one of the indispensable ingredients in the modern feed industry, various additives have obvious effects on strengthening the nutritional value of basic feed, improving animal production performance and improving the quality of livestock products. For example, acidifiers are environmentally friendly additives with no residues, no risk of microbial resistance and no toxicity. As a consequence, they are widely used in livestock and poultry feed both in China and globally. Acidifiers can not only reduce the gastrointestinal pH value and regulate the structure of gastrointestinal microflora but are also directly involved in nutrient metabolism, thus promoting digestion and absorption. Therefore, it also has a definite effect on the development of intestinal villus. A study on acidifiers observed that 0.10%, 0.15% and 0.20% 2-hydroxy-4-methylthiobutyric acid added to the drinking water of broiler chickens significantly increased villus height in broilers by 18% (from 450 μm to 531 μm) [60]. Elhassan et al. also found beneficial effects of acidifiers on broiler gut morphology [61]. As a class of highly effective, non-toxic, side-effect-free and environmentally friendly feed additives, feed enzymes have received increasing attention as catalytic acidifiers. Xylanase [62] and phytase [63] supplementation increased villus height by 15% (from 400 μm to 460 μm) and 20% (from 380 μm to 456 μm) in nursery pigs. In addition, lysozyme (LZ) has natural antimicrobial activity and is being investigated as an alternative to antibiotics. One study used LZ to improve the growth performance and intestinal barrier function of 22-day-old weaned piglets, revealing a tendency toward increased intestinal villus height [64].

## EFFECTS OF AGE ON INTESTINAL VILLUS

The intestinal tract undergoes distinct morphological and functional changes at different life stages, which can be broadly categorized into two distinct processes: developmental maturation (from birth to adulthood) and senescence (age-related functional decline). The following discussion aims to disentangle these two processes and their impacts on villus architecture.

### Developmental maturation

During early life stages, the intestinal tract undergoes adaptive remodeling driven by nutritional transitions and organ development. In precocial species such as goats, villus height initially decreases post-weaning due to stress-induced atrophy but subsequently increases as rumen development enhances nutrient utilization [65]. This “V-shaped” recovery pattern, which has been observed in goat kids, African ostrich chicks [66], hens [67] and the channel catfish [68], reflects a compensatory adaptation to shifting metabolic demands. However, the developmental level and growth rate of villus varies among different species of animals, even at the same stage of development. For instance, male Peking ducks reach market weight faster than turkey chicks despite similar hatching weights and incubation periods. This discrepancy can be attributed to the accelerated intestinal maturation observed in ducks, which occurs within 0 to 3 days post-hatching [69]. This maturation is characterized by rapid villus elongation and crypt expansion, facilitating efficient nutrient absorption and supporting accelerated somatic growth. These adaptations underscore the critical role of developmental plasticity in shaping early-life intestinal morphology.

### Senescence

Aging is a complex physiological process in which most organs decline [70]. In the digestive tract, aging can lead to intestinal barrier disorders and villus degeneration [71]. In particular, the small intestinal villus and crypts decrease in size and number, which impairs the absorptive capacity [72]. Recent studies demonstrate that a reduction in RAB-10 (Member RAS Oncogene Family, is a Protein Coding gene) function in senescent nematodes and mice results in the disruption of adhesion junction structures. This has been shown to result in damage to intestinal villus and a significant decrease in villus height [73]. Age-related dysfunction of intestinal stem cells impairs their regenerative capacity, directly contributing to villus shortening through reduced epithelial turnover [74]. Nicotinamide mononucleotide (NMN), a compound associated with the process of anti-aging, has been observed to significantly increase villus height and improve intestinal barrier function in aged mice by elevating NAD^+^ levels. It has also been demonstrated that NMN can reduce inflammatory responses through the activation of sirtuins (e.g., SIRT3 and SIRT6) and antioxidant genes (e.g., SOD2 and NRF2), thereby reversing aging-associated villus atrophy [75].

## EFFECTS OF DISEASES ON INTESTINAL VILLUS

### Intestinal diseases

Necrotizing enteritis (NE), also known as enterotoxemia, presents as sick and dead chickens with mucosal necrosis of the posterior segment of the small intestine. Once infected, the health status and production performance of poultry can be severely affected [76]. Xu et al found that when constructing an NE model of *Clostridium perfringens* infection, chorionic villus height and the ratio of chorionic villus height to crypt depth were significantly lower in the infection model [77].

Whipple’s disease is a disorder of fat metabolism of intestinal origin. Researchers found significant villus atrophy and moderate crypt hyperplasia in untreated patients, with more than a 50 percent reduction in villus length, combined with an 86 percent increase in crypt length. The data indicated that duodenal villus atrophy and crypt hyperplasia in patients with this disease are associated with apoptosis of epithelial cells and decreased alkaline phosphatase expression [78].

Celiac disease is an inflammatory disorder of the upper small intestine (duodenum and jejunum) in genetically susceptible individuals. In patients with celiac disease, the jejunal mucosa is flattened, lacks a normal villus, and is infiltrated by lamina propria cells with an increased number of intraepithelial lymphocytes. Thus, disruption of the epithelial barrier and atrophy of villus are defining features of celiac disease [79].

Changes in intestinal villus height are not only directly related to pathological damage but also to key signaling molecules that regulate intestinal homeostasis. The Wnt signaling pathway is one of the critical signaling pathways controlling intestinal homeostasis and cancer. C-Jun is a member of the AP-1 family of transcription factors involved in the regulation of cell proliferation, differentiation and apoptosis. In the intestine, c-Jun affects the maintenance of villus structure by regulating the strength of the Wnt signaling pathway. It has been found that C-Jun deficiency leads to a reduction in villus length, whereas c-Jun overexpression promotes villus regeneration [80]. This mechanism is particularly important in diseases such as necrotizing enterocolitis, where villous atrophy is a typical feature of these diseases.

In a similar manner, HuR, a nucleoplasm RNA-binding protein that is widely expressed, has pleiotropic effects on cell growth and tumorigenesis. In mice with gut-specific knockout of HuR, it was found that villus was highly reduced and that there was compensatory migration of proliferating cells in an Adriamycin-induced acute intestinal injury model [81]. This finding suggests an important role for HuR in intestinal injury repair and maintenance of villus structure.

### Rheumatoid arthritis

Rheumatoid arthritis (RA) is an inflammatory autoimmune disease that destroys bone and cartilage, which is also considered a multisystemic disease affecting the gastrointestinal system and other organs. A rat model showed that RA resulted in intestinal inflammation combined with atrophy of the mucosa and intestinal wall, causing an 11% reduction in intestinal villus height [82].

### Traumatic brain injury

Numerous clinical studies demonstrate the existence of certain interactions between the brain and intestinal system, which coordinate with each other to jointly influence key physiological roles and homeostasis of the organism [83]. It has been reported that traumatic brain injury (TBI) could cause significant changes in the structure and barrier function of the intestinal mucosa. Villus height, crypt depth, and surface area decreased markedly at 24 h post-injury, eventually leading to mucosal atrophy on day 7 post-injury. The apoptotic index was highly negatively correlated with villus height, crypt depth, and villus surface area [84].

### Microbial infections and toxins

Porcine epidemic diarrhea virus infection causes both vomiting and diarrhea, especially affecting nursing and weaning piglets [85]. It was reported that microscopic lesions include severe diffuse atrophic enteritis with a significant reduction of villus height [86]. In the early stage, porcine enteric calicivirus and bovine enteric calicivirus were observed to infect the proximal villus enterocytes of the small intestine, inducing villus atrophy and crypt hyperplasia, thereby producing diarrhea in their respective hosts, which severely disrupts the state of health and performance of the organism [87].

Infectious bursal disease, caused by the infectious bursal disease virus, is a disease affecting the worldwide poultry industry. Strongly virulent bursal virus infection was associated with severe histological lesions in the intestine, whereby villus height in the duodenum, jejunum, and ileum was reduced after infection [88].

Mycotoxins are secondary metabolites of filamentous fungi that frequently contaminate cereals and cause significant economic losses in global animal husbandry, among which aflatoxin B1 (AFB1) is arguably the most toxic. Feeding an AFB1-contaminated diet significantly reduced jejunal villus height, villus width, and surface area at 14 d, indicating that animal growth performance and intestinal morphology were adversely affected, whereby digestive physiology and development were altered [89]. In addition to aflatoxin producers, *Fusarium* widely contaminates cereals and maize worldwide. It can produce deoxynivalenol (DON), zearalenone and fumonisin. During the investigation of the effect of feed-derived fusarium mycotoxins on broiler performance, the intestinal villus of broiler chickens fed DON-contaminated diets was consistently significantly lower than those of the untreated group [90].

*Eimeria* infection can lead to the shortening of villus and reduction of the intestinal absorption area, affecting the growth of broilers. The villus parameters of broilers treated with diclazuril demonstrated a positive effect of the agent on intestinal patterns. Treated chickens showed shallower crypts and longer villus compared to the control group [91,92].

## EFFECTS OF DRUGS ON INTESTINAL VILLUS

During the treatment of common diseases, some medications can lead to villus atrophy. For example, the symptoms of spruce-like enteropathy caused by Olmesartan, a drug for the treatment of hypertension, are similar to those of celiac disease, raising the risk of misdiagnosis. Pharmacological enteropathy can be suspected after celiac disease and other causes of villus atrophy are ruled out, with clinical improvement and histological recovery confirmed after discontinuation of the medicine [93].

The antineoplastic drug 5-fluorouracil (5-FU) is one of the most common cytotoxic drugs associated with mucositis that can also affect the height of villus after administration. Chemotherapy-induced gastrointestinal mucositis occurs in approximately 50% to 80% of patients. Some researchers used non-obese diabetic /severe combined immunodeficient mice to model the responses of chemotherapy patients. Their results showed that the administration of 5-FU shortened the mean height of jejunal villus in this model [94].

Indomethacin (IDMT) was one of the first nonsteroidal anti-inflammatory drugs used to treat migraines and headaches, and its common adverse effects are also related to the gastrointestinal tract [95]. A study using IDMT to induce intestinal inflammation in newborn pigs revealed thinning of the intestinal wall as well as ulceration of the distal jejunum and ileum. In addition, IDMT reduced the height of the intestinal villus and the surface area of the villus in the jejunum of piglets [96].

Bowel resection is a surgical intervention that removes part of the intestines and is used to treat diseases or obstructions of the large intestines. Notably, bowel resection also influences the height of intestinal villus. Investigators explored the effects of altered glutathione redox status on the ileal mucosa after massive small bowel resection (SBR) in a rat model of short bowel syndrome. It was found that SBR stimulated adaptive increases in ileal villus height and total mucosal height on postoperative day 7 [97]. STAT1 is a pivotal signal transducer and transcriptional activator that plays a crucial role in the regulation of immune responses and cell proliferation. In certain intestinal diseases, aberrant activation of STAT1 has been observed to result in increased inflammatory response and destruction of villus structure. Inhibition of the STAT1 signaling pathway can attenuate intestinal inflammation and promote villus repair [98].

In most situations, ischemia-reperfusion injury is followed by recovery of tissue and organ function, repair of damaged structures, and improvement in the patient’s condition. However, sometimes ischemia-reperfusion injury aggravates the dysfunction and structural damage of tissues and organs. A significant increase in the intestinal crypt depth histomorphometry was observed in the early stages of reperfusion, followed by an increase in the height of the intestinal villus. After 24 hours of reperfusion, intestinal villus was 15% longer than in untreated controls, significantly increasing the height of intestinal villus after 24 hours of reperfusion [99].

The use of medication and other treatments related to the intestinal tract can have different degrees of negative impacts on the height of intestinal villus. Therefore, prevention of the disease as early as possible after the onset of the disease and appropriate therapeutic measures to minimize damage to the intestinal villus are mandatory.

## EFFECTS OF ENVIRONMENTAL FACTORS ON INTESTINAL VILLUS

The rearing environment is one of the most important considerations in farm management. The light, temperature, stocking density with its related physical stress and other factors in the husbandry environment will have a major or minor impact on the performance indicators of farmed animals. Different environmental conditions during feeding have different degrees of influence on the organism, and thus on the height of intestinal villus.

First, light in the breeding environment has an important influence on the height of intestinal villus. It was found that the intestinal villus of broilers in each monochromatic light illumination group had better integrity, and the height of intestinal villus gradually increased throughout the growth stage, but it was dissimilar in different illumination groups. Broilers in the red-light group had the lowest height of intestinal villus. On days 7 and 21, the green light group had significantly higher villus heights than the white light group. By day 49, the blue light group had the greatest villus height [100]. Therefore, husbandry managers can provide livestock and poultry with different illumination and feeding stages to improve animal production performance.

Temperature is another crucial factor in the environment. Barri et al demonstrated an interaction between temperature and age on duodenal villus height during incubation, with temperature being the most significant factor. Broilers raised at a high temperature (39.6°C) displayed increased villus height [101]. However, duodenal villus height decreased linearly with increasing environmental temperature (18°C, 21°C, 24°C, 27°C, and 30°C) in 28 to 49 day-old geese [102]. Therefore, for various animals, different rearing stages must ensure that appropriate temperatures are strictly controlled and that performance indicators are closely monitored to ensure that they are in the optimal range.

The density of rearing also needs to be emphasized in the husbandry management process. High stocking density adversely affects the length and number of intestinal villus as well as the size of goblet cells. It was found that the medium and high stocking density groups had the lowest villus height compared to the low stocking density group. Although muscle thickness, villus thickness, and villus strength did not significantly differ between stocking density groups, villus height decreased significantly with increasing stocking density [68].

Another influential factor is physical stress, including heat stress, weaning, severe trauma, tail biting, and similar adverse stimuli. Gogoi et al found that the villus height of the ileum was significantly affected by body weight, duration of heat stress, and their interactions. Intestinal villus height increased with the duration of heat stress, and the length of villus was higher in the lightweight broiler group than in the medium- and high-weight groups [103]. Severe trauma causes impairment of intestinal mucosal integrity and function, leading to a decrease in small intestinal epithelial cell proliferation and apoptosis. In addition, the symptom of tail biting is a clinical presentation of porcine stress syndrome, which is a non-specific stress response caused by many types of adverse factors that stimulate pigs under modern husbandry regimes. The findings of Palander et al showed that in the proximal and mid-jejunum, the height of the villus was greater in non-biting than in tail-biting pigs, indicating a greater ability to absorb nutrients in the proximal jejunum. Furthermore, pigs in tail-biting barns exhibited decreased jejunal villus height and blood amino acid levels, due to the decreased absorption capacity, feeding behavior, as well as tail-biting stress [104]. Weaning stress is another physical stress related to nutritional stimulation. The shift in nutrition source leads to structural and functional damage to the intestine, which in turn affects the height of intestinal villus. A study showed that weaning stress reduced duodenal villus height by 28% in the three-day weaning group, 16% in the seven-day weaning group, with values of 34% in the jejunum and 17% in the ileum compared to the lactating piglet group [105]. Therefore, it is vital to pay attention to the state of animals to eliminate the negative impact of the feeding environment due to changes in the external environment as early as possible.

## CONCLUSION

**Figure 1:**
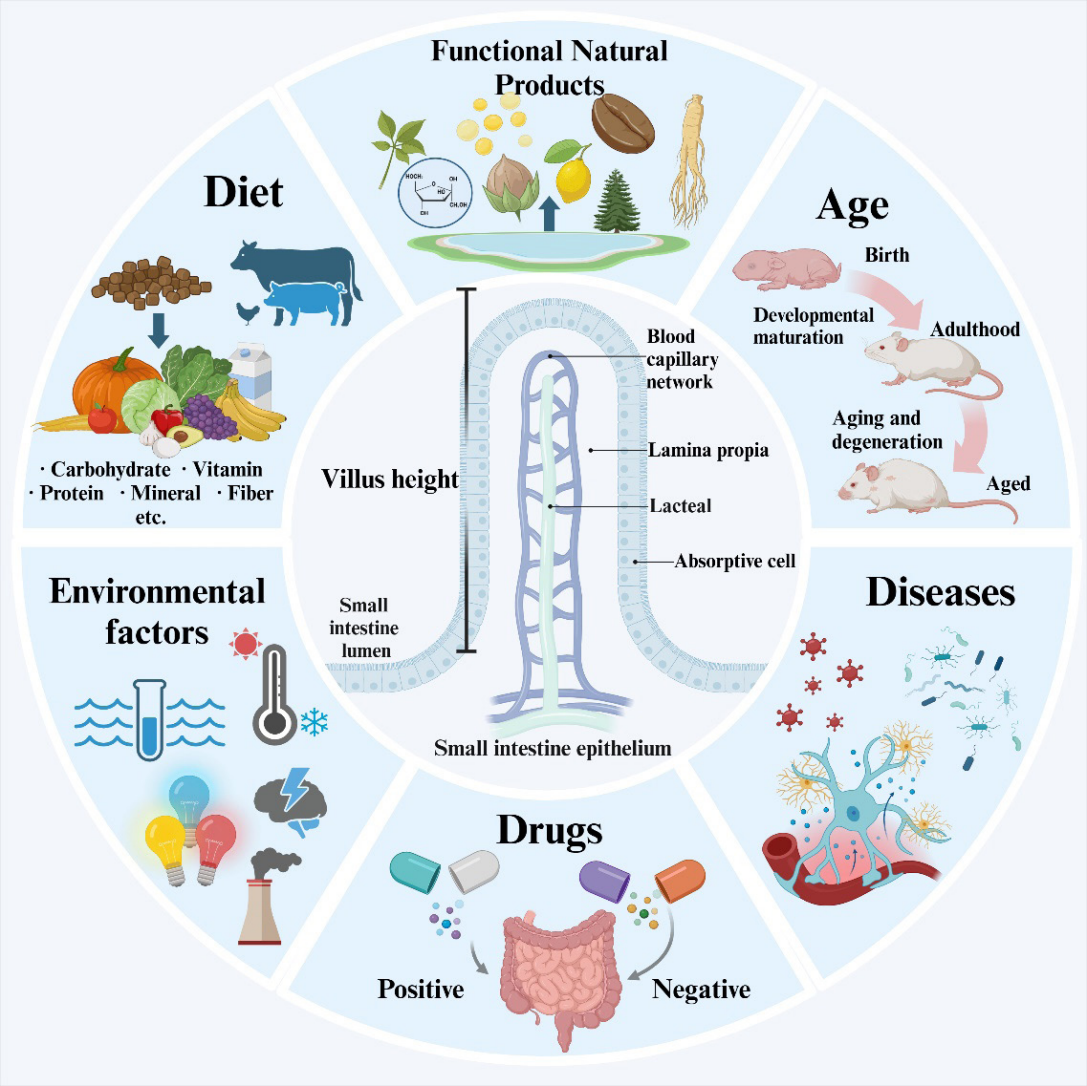
Influencing factors of intestinal height.

Taken together, as shown in Figure 1, this review summarizes the common factors affecting the height of intestinal villus in humans and animal models: diseases, nutritional deficiencies and environmental factors can all cause atrophy of intestinal villus, resulting in intestinal microenvironmental disorders. An optimized diet, supplementation of nutrients, improvement of environmental conditions and timely prevention of epidemics can effectively increase the height of villus and further improve intestinal health. In order to optimize the protection of the intestinal tract of both humans and farm animals, it is imperative to allocate greater attention to these factors in both daily life and animal husbandry.
